# 20-Year trends in the social participation of the oldest old

**DOI:** 10.1177/14034948241261720

**Published:** 2024-08-08

**Authors:** Erika Augustsson, Stefan Fors, Johan Rehnberg, Carin Lennartsson, Neda Agahi

**Affiliations:** 1 Aging Research Center, Karolinska Institutet and Stockholm University, Stockholm, Sweden; 2 Department of Public Health Sciences, Stockholm University, Stockholm, Sweden; 3 Centre for Epidemiology and Community Medicine, Region Stockholm, Sweden; 4 Swedish Institute for Social Research, Stockholm University, Stockholm, Sweden

**Keywords:** Trends, social participation, oldest old

## Abstract

**Aims:**

To investigate 20-year trends in social participation among the oldest old (77+ years) in Sweden and assess the extent to which changes in educational attainment and functional abilities explain these trends.

**Methods:**

Seven waves of the Swedish Panel Study of Living Conditions of the Oldest Old (SWEOLD) spanning 2002–2021 were used with a repeated cross-sectional design. To analyse the association between time and social participation we employed the Karlson–Holm–Breen method of decomposition. The study focused on informal social participation (contact with friends and family), leisure participation (public or semi-public gatherings), and formal participation (organisational engagement and study circle attendance).

**Results:**

Both leisure and informal participation peaked in 2014 and declined in 2021, influenced by the COVID-19 pandemic, whereas formal participation showed a slight increase in 2021. Total participation increased at least until 2011. Overall, older adults have increased their levels of social participation in recent decades, disregarding the influence of the pandemic. Decomposition analysis revealed that population-level changes in educational attainment and functional abilities explained a substantial portion of the observed trends.

**Conclusions:**

**As the proportion of older adults continues to rise, it becomes increasingly important to understand the developments and drivers of behavioural change in the older population. As more people are socially active, there may be increasing differences between those participating and those not – which could lead to increased inequalities. The observed trend in increasing participation, influenced by changes in education and health, emphasises the importance of fostering age-friendly environments and addressing potential social inequalities among older adults.**

## Background

The proportion of older adults in Sweden and Europe is increasing rapidly, with a projected one-third of the European Union (EU) population aged over 65 years by 2050 [1]. The World Health Organization has identified social participation as one of the key pillars of an age-friendly community [2]. It plays an important role in reinforcing social relationships, social support, and social integration, which can result in improved overall life satisfaction, higher quality of life and enhanced psychological wellbeing [3]. Social participation can be defined as a person’s involvement in activities providing interactions with others in community life and in important shared spaces [4]. Structural changes and demographic shifts have likely led to changing patterns and opportunities for older adults to participate in various types of social activities [5]. This study aims to explore 20-year trends in social participation among the oldest old (77+ years) in Sweden, investigating to what extent changes in educational attainment and functional abilities explain these trends.

### Defining social participation

We adopt the social interaction perspective [6] when discussing social participation, focusing on social connections. Van Ingen [7] identified two main aspects of social participation. Formal social participation involves membership and engagement in voluntary associations [8, 9]. Conversely, informal social participation lacks fixed membership, being more self-organising, spontaneous and flexible [8]. We align with previous research by examining informal social participation components, specifically social contact with friends and family (hereby called informal social participation) and public or semi-public social gatherings such as visits to restaurants and cultural events (hereby called leisure participation) [7, 10, 11]. Examining these multiple forms of social participation allows us to assess the extent of changes in social participation levels.

Research conducted in Sweden has demonstrated the significant value that older adults place on formal social participation [12]. Engaging in these activities is seen as a source of diversity and meaning in life, providing individuals with a sense of continued contribution to society and fostering social involvement.

### Previous findings

A study spanning 2004 to 2017 indicated a slight increase in formal participation among older adults across Europe, with persistent inequalities in access to these activities [9]. Sweden had the highest level of formal participation and the lowest relative differences in participation of older people between different groups (e.g. by education or health status). In the Netherlands, persons in the year 2000 exhibited lower informal social participation but increased leisure participation compared with persons 25 years earlier, suggesting shifting preferences in how to socialise [7]. Also in the Netherlands, formal and leisure social participation increased among 60–69 year olds from 1992 to 2002, but the positive impact of higher education in the later cohort was offset by poorer health status, highlighting cohort-specific differences [8].

In the United States, Ang [13] identified a different pattern in the population, characterised by an increase in formal social participation among later-born cohorts, while informal social participation remained relatively stable. Similarly, there has been a reduction in the incidence of older adults’ exclusion from social relations and leisure in Sweden over the past decades, indicated primarily by increasing leisure participation between 1992 and 2011 [14].

### Potential drivers of changes in social participation

Increasing individualistic values and practices over the past decades may have led older adults to have more freedom and autonomy in their pursuits of social interests and preferences [15]. The opportunities for post-retirement participation in society have changed, influenced in part by improvements in health status and changing perceptions of older adults’ capabilities. In Sweden, successive generations of older adults have seen a gradual increase in functional capacity across various dimensions, including physical, sensory and cognitive, as well as a decreasing prevalence of depression [16, 17]. Higher physical function potentially contributes to cohort differences in social participation due to health-related changes. In addition, increased life expectancy delays widowhood, and having a partner has been shown to increase the likelihood of social participation [8].

For cohorts born during the first half of the 20th century there has been a gradual increase in education and employment levels, especially among women [18]. Higher socioeconomic status, especially educational attainment, has a positive association with most types of social participation as well as more active social profiles in later life [19]. This can be for many reasons; higher socioeconomic status is associated with better health status and more engagement in wider ranges of activities outside the family. One study which specifically looked at social participation in older adults (85+ years) found that the number of activities, although including solitary activities, was higher in those with higher educational attainment and intact walking ability [20]. Increased access to education, and through that the labour market, may provide the means to socialise both through higher income and increased social contacts.

### The current study

There is a distinct lack of knowledge about the development of social participation of older adults over time. Most studies are at the overall population level, have an upper age limit, or compare only two data points, which may mask fluctuations over time. In this study, we explore 20-year trends in social participation of the oldest old (77+ years) in Sweden and ask to what extent educational expansion and decreasing prevalence of disability may account for these changes. In addition, we look at whether we see different trends for different components of social participation. With this study, we address the need to understand the shifting dynamics of social participation among older adults. With the proportion of older adults rapidly increasing, there is a demand for insights into fostering age-friendly environments.

## Methods

### Design and participants

The Swedish Panel Study of Living Conditions of the Oldest Old (SWEOLD) is a national survey conducted repeatedly since 1992. SWEOLD is based on random samples of the Swedish population aged around 75 years and above, including persons living in institutions. To make the waves comparable we set 77 as the lower limit as that was the lowest age represented in all waves. To increase the statistical power in the oldest age groups, later waves of SWEOLD have been supplemented with an oversampling of groups aged 85 years and older. In 2021 an additional sample from Dalarna County was added, weights are used to account for both the oversampling of the oldest old, and the oversampling of residents of Dalarna. This study used waves from 2002, 2004, 2011, 2014 and 2021 to cover approximately 20 years, 1992 was not used due to missing data on social participation. The response rate ranged from 84.4% in 2002 to 64.0% in 2021. This study used a repeated cross-sectional design. The final sample consisted of 3731 participants who had data on at least one participation variable. The respondents spanned birth cohorts born between 1903 and 1944, aged between 77 and 109 years with the same lower age limit each year.

In 2002 and 2011 direct face-to-face interviews were conducted while interviews were carried out over the phone in 2004, 2014 and 2021. To be able to include frail and cognitively impaired individuals in the sample, proxy interviews were conducted with a close family member or someone who knew the respondent well if the respondents were unable to participate themselves due to health reasons. All respondents gave their consent to participate.

Ethical approval for the study has been granted by the Swedish Ethical Review Authority (2019-06324; 2021-00393). For more detailed information about the SWEOLD study, see Lennartsson et al. [21].

### Social participation

Information on formal social participation was obtained through self-reported organisational engagement and study circle attendance (for details on questions and response options for all social participation variables see Supplemental Table I). People who participated in organisations or associations at least a few times a year or study circles sometimes or often were coded as having formal participation. Information on formal social participation was only available in 2002, 2011 and 2021.

Informal social participation was measured as visiting or being visited by friends and the same two questions were asked for relatives. Response options ranged from (0) never to (2) often. The interviewers were directed to tell the participants not to include children as relatives in the data. These four variables were summed and a score of 4 or above was classified as informal participation.

Leisure participation was measured using two variables capturing attending cultural events and dining out. If respondents indicated that they did one or both activities at least sometimes they were classified as participating in leisure activities.

To attempt to account for the impact of COVID-19 in 2021, the option ‘no, because of the pandemic’, which was available for informal and leisure participation, was coded as ‘sometimes’ in a ‘covid-adjusted’ variable. Questions regarding the frequency of attending meetings with organisations had no response options related to the pandemic.

A variable for any social participation was measured as participating in at least one of formal, informal or leisure participation. As formal participation is only measured in three periods, so is this participation indicator.

### Education and functional ability

Education was measured by self-reported highest attained education, distinguishing between those who had achieved compulsory education or below and those with a secondary/tertiary education level.

A measure of functional ability was created for this study by applying principal component analysis to three commonly used indices measuring different aspects of function. Mobility limitations were measured through the ability to run 500 m, walk 100 m and walk up some stairs without difficulty, giving an index between 0 and 3. Instrumental activities of daily living (IADL) were used to indicate functional capacity. The ability to, without help, purchase food, cook, and clean was self-assessed and created an index between 0 and 3. Finally, activities of daily living (ADL) were measured by the ability to get in and out of bed, get dressed, go to the bathroom on their own, eat, and wash their hair, leading to an index between 0 and 5.

The results of the principal component analyses were supported using confirmatory factor analysis which accounts for clustering with a 0.996 correlation, and a Cronbach’s alpha test of 0.8. It was further standardised with Z-scores for easier interpretations.

### Time

Time was measured as a categorical variable, with one category per year of data collection.

### Statistical analysis

First, descriptive proportions were presented to benchmark the trends in social participation, education, and functional ability. Second, the association between time and participation were analysed with logistic regressions using the Karlson–Holm–Breen (KHB) method of decomposition [22]. Robust standard errors were used alongside the clustering of non-independent observations across the study waves to account for repeated measures.

### Decomposition analysis

Although mediation analysis is more commonly used to analyse the impact of intermediate variables on a causal effect of an exposure on an outcome, it is used and interpreted differently in this study, similar to Badache et al. [16]. The KHB method allows for comparison between nested models which, due to the non-collapsibility of odds ratios, normally cannot be done when nested non-linear models are calculated separately [22]. This method compares the regression coefficients for the outcome variable (social participation) from two models. The crude and adjusted coefficients are measured on the same scale and are thereby unaffected by the rescaling bias that comes from cross-model comparisons of non-linear models [23].

The KHB output shows the estimated effect of the reduced model (total effect), the full model (direct effect), and the estimated differences between these two (indirect effect). The reduced model shows the total effect of time on social participation. The full model shows the direct effect of time that is left after controlling for educational attainment and functional ability, or the residual unexplained variance in the time trend. Finally, the difference between the reduced and full model shows the indirect effect of time on social participation, the part that could be attributed to compositional changes in the distribution of educational attainment and functional ability in the population.

The KHB model also calculates a ratio between the total time effect and the direct effect as well as a percentage indicating how much of the total time effect could be attributed to increases in educational attainment and functional ability over time. A confounding percentage exceeding 100% indicates that without the observed changes in the distribution of education and function, the development would have gone in the other direction. In our case, that would mean that without increasing education and improved functional ability, social participation would have decreased rather than increased. Yet, such an interpretation should be validated by carefully checking the confidence intervals for the direct effects [16].

The results are reported as average partial effects (APEs), giving the decomposition a more substantial interpretation than the odds ratio. APEs are measured on the probability scale and estimate the average marginal effect of time on social participation in the population [23].

All analyses were completed in Stata 17.0.

## Results

Table I shows the descriptive data of the sample. The latest wave has a lower proportion of people with indirect interviews and living in institutions, this is likely due to a combination of a lower response rate and the proportion of people living in institutions declining since 2010 [24]. Preliminary attrition analyses indicate slightly higher retention of persons with higher education and no significant evidence of systematic attrition related to health in 2021. There are more women than men in each wave, which reflects the population composition [25]. The proportion of married participants increases each wave which likely reflects increased life expectancy. The mean age across all waves is around 83 years.

**Table I.** Descriptive statistics in percentages.

Year	2002	2004	2011	2014	2021	2021^a^
Sample characteristics						
Proxy interview	20.8	21.4	20.1	17.9	14.7	
Living in an institution	14.8	10.5	11.7	13.0	7.4	
Women	59.2	61.0	62.1	58.9	58.5	
Married/partnered	35.6	38.2	44.7	47.3	50.7	
Age – mean (SD)	83.3 (4.9)	83.3 (4.8)	83.4 (5.0)	83.2(5.0)	83.5 (5.2)	
Societal developments						
Post-compulsory education	31.8	35.1	42.3	48.2	65.1	
Functional ability – mean (SD)	–0.15 (1.1)	–0.06 (1.0)	–0.04 (1.0)	0.05 (1.0)	0.22 (0.9)	
Social participation						
Formal	42.6	–	38.8	–	47.3	–
Informal	57.3	53.2	62.3	63.2	43.5	63.2
Leisure	45.9	49.4	63.3	64.7	50.6	68.4
Social participation	69.7	–	80.2	–	73.5	82.3^b^
N – not weighted	621	648	904	722	1322	

All proportions are weighted.

SD: standard deviation.

a People who answered ‘No, because of the pandemic’ are coded as doing the activity. This was not possible for formal social participation.

b Participating in any of the two adjusted or formal types of social participation.

**Figure 1.**
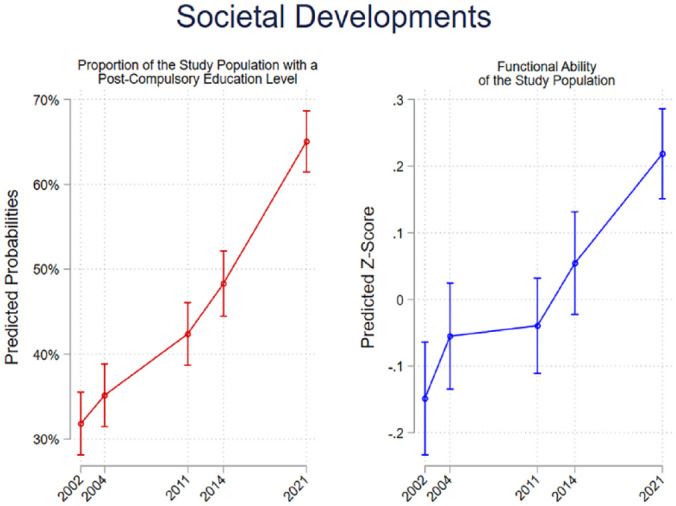
Predicted probabilities of higher education and predicted levels of functional ability across waves.

Figure 1 shows the predicted probability of the population having reached secondary education or above as well as the predicted functional ability of the population. The proportion of older adults with higher education increased over the period, as did the level of functional ability which improved continuously throughout the study period.

**Figure 2.**
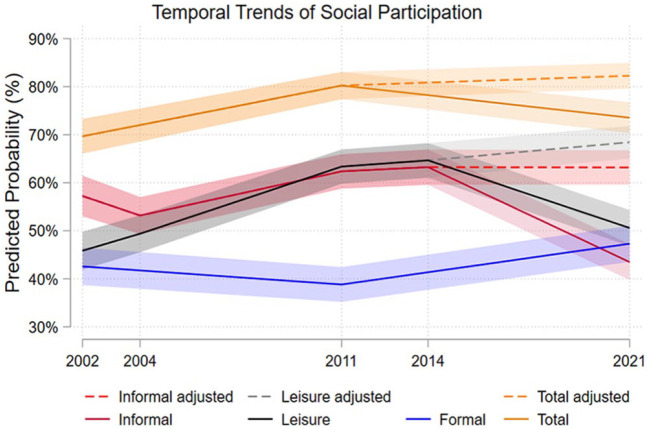
Predicted probabilities of social participation 2002–2021 age 77+ years in Sweden, adjusted = adjusted for behavioural changes due to the COVID-19 pandemic.

Figure 2 illustrates the changes in social participation over the study period. Informal and leisure participation both increased up to 2014 and then decreased. The COVID-19 pandemic had a clear impact on older persons’ social participation. Around 45% of adults aged 77+ years had informal social participation in 2021, and a slightly higher proportion engaged in leisure social participation. If those who did not do these activities because of the pandemic had done so, the increasing trend would have continued and around two-thirds of older adults would have participated in leisure or informal social activities. Formal participation remained relatively stable and increased in 2021.

### Decomposition of the time trend

Table II reports the results of the decomposition of the trend in social participation to assess to what extent the changes can be attributed to changes in the composition of the population in terms of educational attainment and functional abilities. We only interpret the estimates from the model when the total trend and the decomposed trend are statistically significant. As we are primarily interested in the long-term trends, we focus on the results for 2002–2021. As the variable for 2021 assumes that those who did not participate because of the pandemic would otherwise have done so, we also present the trend to the closest data point before the pandemic (i.e. 2014). This allows comparisons with the shorter trend (2002–2014), which does not make any assumptions about participatory behaviour. All years are presented in Supplemental Tables II and III.

**Table II.** Decomposition of trends in social participation among older adults (77+ years) in Sweden since 2002, with and without adjustments for composition of educational attainment and functional ability.

	Leisure		Informal		Formal		Any	
	2002–2014	2002–2021	2002–2014	2002–2021	2002–2011	2002–2021	2002–2011	2002–2021
	OR		OR		OR		OR	
Reduced (total)	**2.89**	**3.25**	**1.35**	1.27	0.89	**1.33**	**2.24**	**2.34**
	2.20, 3.78	2.50, 4.23	1.04, 1.78	0.98, 1.64	0.70, 1.13	1.04, 1.70	1.66, 3.03	1.74, 3.14
Full (direct)	**1.96**	**1.59**	1.11	0.87	**0.75**	**0.76**	**1.81**	1.17
	1.50, 2.56	1.23, 2.06	0.85, 1.46	0.66, 1.13	0.59, 0.95	0.59, 0.98	1.34, 2.44	0.87, 1.58
Difference (indirect)	**1.47**	**2.04**	1.22	**1.46**	**1.19**	**1.74**	**1.24**	**2.00**
	1.14, 1.90	1.57, 2.66	0.98, 1.52	1.16, 1.84	1.01, 1.39	1.46, 2.09	1.02, 1.49	1.62, 2.47
	APEs		APEs		APEs		APEs	
Reduced (total)	**20.3**	**22.3**	**6.1**	4.7	–2.4	**5.9**	**10.4**	**11.6**
	15.3, 25.3	17.5, 27.1	0.8, 11.4	0.5, 9.9	–7.3, 2.5	0.8, 11.0	7.1, 15.2	7.6, 15.7
Full (direct)	**12.7**	**8.9**	2.1	–2.9	**–5.9**	**–5.1**	**7.6**	2.2
	7.7, 17.7	3.9, 13.9	–3.2, 7.4	–8.2, 2.5	–10.8, 1.1	–10.8, –0.3	3.8, 11.4	–1.9, 6.3
Difference (indirect)^a^	7.6	13.4	4.0	7.6	3.6	11.5	3.6	9.4
	Confounding		Confounding		Confounding		Confounding	
Ratio	1.6	2.5	2.9	–1.6	0.4	–1.0	1.4	5.3
Percentage	36.5	60.6	65.2	160.7	–148.4	195.2	26.7	81.3
	% Mediated		% Mediated		% Mediated		% Mediated	
Education	16.6	29.3	7.1	18.3	–47.9	64.7	10.0	31.2
Functional ability	19.9	31.3	58.2	142.3	–100.5	130.5	16.8	50.1

Numbers in bold are significant at the 95% level.

OR: odds ratio; APEs: average partial effects.

a 95% Confidence interval cannot be calculated because standard errors of indirect effects are unknown for the APE method.

The average likelihood of leisure participation increased significantly over time (odds ratio (OR) 3.25; 95% confidence interval (CI) 2.50–4.23). After including compositional changes in education and function, the direct time effect between 2002 and 2021 decreased to OR 1.59 (CI 1.23–2.06). Or, when expressed through average partial effects, the predicted probability of leisure social participation increased by 22.3 (CI 17.5–27.1) percentage points between 2002 and 2021. After controlling for compositional changes in educational attainment and functional ability, this increase was reduced to 8.9 (CI 3.9–13.9) percentage points (direct effect), leaving an increase of 13.4 percentage points attributable to changes in education and function. As the confounding ratio and confounding percentage indicated, the total time effect was 2.5 times larger than the direct effect, and 60.6% of the total time effect could be attributed to increases in educational attainment (29.3%) and functional ability (31.3%) over time.

The predicted probability of informal social participation increased between 2002 and 2021 (OR 1.27; CI 0.98–1.64/APEs 4.7, CI 0.5–9.9), although this increase was statistically non-significant. On the other hand, the increase between 2002 and 2014, which was unaffected by the pandemic, was statistically significant (OR 1.35; CI 1.04–1.78/APEs 6.1, CI 0.8–11.4). The confounding ratio for 2002–2014 suggests that the bulk of the change (65.2%) could be attributed to changes in education and functional abilities, but as the decomposed effect estimates were statistically non-significant, these patterns should be interpreted with caution.

The probability of formal participation decreased between 2002 and 2011 (OR 0.89; CI 0.70–1.13/APEs −2.4, CI −7.3–2.5) but saw an increase between 2002 and 2021 (OR 1.33; CI 1.04–1.70/APEs 5.9, CI 0.8–11.0). The confounding percentage for 2002–2021 greatly exceeds 100%, which suggests that the trend would have gone in the other direction if it was not for changes in education and functional abilities.

The overall probability of any social participation in old age has increased over time. The predicted probability of social participation increased by 11.6 (CI 7.6–15.7) percentage points between 2002 and 2021. After controlling for compositional changes in educational attainment and functional ability, this increase was reduced to 3.6 percentage points (direct effect) and was no longer statistically significant, leaving an increase of 9.4 percentage points attributable to changes in education and function. The total change over time was 5.3 times larger than the remaining change after adjusting for education and function, and 81.3% of the overall change could be attributed to increases in educational attainment (31.2%) and functional ability (50.1%) over time.

Sensitivity analyses using 2004 as the baseline for leisure and informal social participation showed similar results as using 2002 (see Supplemental Table III), although the changes attributable to education and function were no longer greater than the overall change for informal participation between 2004 and 2021; 69.5% of the increase in informal social participation between 2004 and 2021 could be attributed to increases in educational attainment (8.9%) and functional ability (60.7%).

Additional analyses stratifying the population by sex showed that women saw bigger increases in leisure participation than men (26.4 (CI 20–33) vs. 16.7 (CI 9–24) percentage points) while only men experienced increases in formal participation (Supplemental Tables IV–VII). Changes in education and functional ability explained more of the increase in leisure participation for men (education: 36.6%; functional ability: 43.7%) than for women (education: 26.2%; functional ability: 25.9%). Overall, the participation indicator increased roughly equally between women and men (10.8 (CI 5–16) vs. 12.7 (CI 7–19) percentage points); however, women’s increased participation could almost fully be attributed to increased educational attainment and functional ability (38.4% and 57.8%, respectively) as opposed to men in whom 65.6% of the increase could be attributed to these factors (24.8% and 40.8%).

Stratification by marital status show higher levels of social participation for married people than unmarried people, although unmarried people may have a slightly higher increase in most types of social participation over time (Supplemental Figure 1).

## Discussion

Our study contributes to the understanding of the changing nature of social participation of older adults by exploring 20-year trends in social participation among the oldest old (77+ years) in Sweden. We additionally investigated the role of changing educational attainment and functional abilities in explaining these trends. Overall, social participation among the older population has increased over the past 20 years, with a decrease in 2021 in all but formal social participation. We used an adjusted variable to assess what trends may have looked like without the pandemic, but it is important to note that the actual levels of participation decreased in 2021. As we found considerable behavioural changes and no decreases in health in 2021, it will be crucial to investigate whether these changes have been sustained or whether people have returned to pre-pandemic levels of social participation. There could be considerable impacts on health and wellbeing if these new, less participatory, behaviours continue.

Components of social participation have changed in different ways over time. The increases in leisure participation, encompassing activities such as restaurant visits and cultural events, echo findings from the Netherlands that identified changing preferences in socialising behaviours [7]. In Sweden, there seems to be a similar trend of older persons engaging more in social activities outside the home. Alongside these changes, the variety and availability of restaurants has increased and the economic standing of older adults as a group has increased, allowing easier access to these types of activities.

Informal social participation, or visits with friends and relatives, has increased only slightly up to 2014. It has been suggested that while patterns of social behaviour may differ across cohorts, social connectedness with friends and family remains largely the same [13]. These interactions are important for the health and wellbeing of older people [26]. A significant proportion of the older population did not engage in informal participation in this study. In particular, over 30% of individuals at any time point did not meet the informal participation threshold (minimum 4 on an 8-point scale). However, the level of informal participation would likely be higher if visits with children had been included, but the high proportion of people with low informal participation is a concern for experiences of social isolation and feelings of loneliness.

Improvements in the health of older adults may lead to older persons increasing their time spent on activities other than informal social participation. Older adults continue to value and maintain their core social connections but may be choosing to invest more of their time in recreational and cultural pursuits, thereby contributing to the observed rise in leisure participation before the pandemic.

In contrast to the fluctuations in informal and leisure participation, formal social participation remained relatively stable over the years. Previous studies have explored the benefits of formal social participation for older individuals, emphasising its role in providing a sense of purpose and continued contribution to society [12]. Interestingly, formal participation was the only type that increased during the last wave. It could be that people turned to formal social activities through online gatherings or organised activities which allowed for safe engagement while limiting their visits with others and their social leisure activities.

The decomposition analysis revealed that a significant portion of the observed trends in social participation could be attributed to changes in educational attainment and functional ability. Compositional changes in these factors explain part of the increasing trend in leisure participation, in roughly equal amounts. High functional ability allows for freedom of movement which in turn means easier access to various spaces. Educational attainment affects income and is a determinant of cultural taste and norms [27], which in turn influence various forms of social engagement. Education not only increases skills and pro-social behaviours [8] but also impacts individuals’ access to the labour market, potentially shaping their social opportunities through expanded networks and contacts. As more people have higher education levels in more recent years there may be increasing inequalities between those with more and less education. There is some evidence for socioeconomic inequalities in social participation increasing in older ages and over time [28].

For all but leisure participation, a larger part of the changes in social participation could be attributed to increasing functional abilities than to educational attainment. The significance of functional ability for social participation highlights the need to lower the barriers to participation to allow people with lower functional abilities access. The availability of activities and organisations geared towards older adults have also been increasing with time. However, the observed increases in formal participation could primarily be attributed to increasing levels of functional ability, thus there may previously have been a desire to partake in these types of activities but not the right level of access.

As women experienced more significant increases in leisure participation while men only experienced increasing formal social participation, there may be differences and changing perceptions of what kind of activities to engage in in old age dependent on sex. Increases in the indicator of participation were almost fully attributable to increased educational attainment and functional ability for women. Decreasing sex differences in educational attainment and functional abilities may thereby have had a great impact for women in many areas, including social participation. Married people were more socially active, although unmarried people may have experienced a greater increase in levels indicating developments in which changing norms around participation may be affecting single people to a greater degree.

Despite an overall increase in participation up to 2014, a substantial proportion of individuals aged 77 years and above in Sweden do not participate in these social activities. This can likely be attributed to a combination of factors, including barriers to access such as physical limitations or financial constraints, and a conscious choice by some older adults to prioritise other aspects of their lives [29]. While lack of participation may be influenced by individual preferences, it is important to recognise the potential consequences. Social isolation and loneliness are linked to a lack of social participation and are associated with worse mental and physical wellbeing among older adults [30]. Addressing this issue requires a comprehensive approach that includes creating age-friendly environments, improving transportation options, and promoting diverse and accessible activities to encourage a more inclusive and fulfilling life for older adults.

### Strengths and limitations

One of the major strengths of this study is the use of a representative sample of the oldest old. Capturing both people living in institutions and people with dementia enhances the robustness and generalisability of the findings to older adults aged 77+ years as opposed to studies with upper age limits or community-dwelling samples. The longitudinal nature of the data, spanning two decades, provides a comprehensive view of trends in social participation among older adults in Sweden.

The declining response rate may introduce a potential source of bias, as non-response could be related to specific characteristics that are associated with social participation, potentially affecting the generalisability of the findings. Particularly in 2021, there may be a risk of more vulnerable people not participating in the survey, although preliminary attrition analyses (not shown) indicate no significant evidence of systematic attrition related to mobility limitations, self-rated health, or depression.

To adjust for consequences of the COVID-19 pandemic, we have used a covid-adjusted variable which assumes that people who responded that they do not partake in the activity due to the pandemic would have done so otherwise. We cannot know how people would have acted had there not been a pandemic, and we also do not make any adjustments for people who may have changed their habits in other ways due to the pandemic.

The study relies on highly subjective measures, the response options ‘sometimes’ and ‘often’ may be interpreted differently by different people and for different activities. However, a strength in this is that we do not decide based on our assumptions on which frequency constitutes participation in the leisure and informal variables. The activities included in leisure participation are limited and may be more attractive and accessible to people with certain levels of financial resources, thus our findings regarding how compositional changes in educational attainment explain these increases may be related to the activities included in the measure. Future studies should aim to include a more varied assortment of activities and include more factors that are associated with participation and have changed over time, such as marital status.

When we estimate the proportions of the observed changes in social participation that can be attributed to increased education and better physical function, the ‘attribution’ should be interpreted in statistical terms. That is, based on these analyses we cannot say that these estimates are due to the causal effects of education and physical function on social participation. It is quite possible that the attributed effects are, wholly or partly, confounded by other factors not in our models. Future studies are needed to test thoroughly the causal assumptions of the model.

Formal participation, having fewer measurement points and lacking COVID adjustments, poses a limitation. Interestingly, it increased between 2011 and 2021 as opposed to the other types of participation which decreased in this time – which may mean that formal participation was more accessible during this time or that the way people engaged with this type of participation differs from the other types.

## Conclusions

At large, social participation increased over time among older adults aged over 77 years up to the pandemic. This rise can partly be linked to more people reaching higher educational levels and maintaining good physical function into old age. However, as more people are socially active, there may be increasing differences between those participating and those not participating which could lead to increased inequalities. To address these gaps, it is important to find out why some participate more than others. Understanding these factors is key to promoting equal opportunities and reducing social inequalities in social activities.

## Supplemental Material

sj-docx-1-sjp-10.1177_14034948241261720 – Supplemental material for 20-Year trends in the social participation of the oldest old Supplemental material, sj-docx-1-sjp-10.1177_14034948241261720 for 20-Year trends in the social participation of the oldest old by Erika Augustsson, Stefan Fors, Johan Rehnberg, Carin Lennartsson and Neda Agahi in Scandinavian Journal of Public Health
