# Supplementary material for: 20-Year trends in the social participation of the oldest old
Source: Scand J Public Health. 2024 Aug 8;53(7):721–30. doi: 10.1177/14034948241261720 (PMC12598059; doi:10.1177/14034948241261720)
Supplement: sj-docx-1-sjp-10.1177_14034948241261720 – Supplemental material for 20-Year trends in the social participation of the oldest old [file sj-docx-1-sjp-10.1177_14034948241261720.docx]

| Table S1. Social participation variables | | |
| --- | --- | --- |
| Formal | Informal | Leisure |
| Do you belong to any society or organisation?   1. Yes 2. No   If yes, Approximately how often do you participate in any such organised activity?  responses ranging from   1. Never or almost never 2. A few times a year 3. Once a moth 4. Once a week 5. Several times a week | Which of the following activities do you usually do: Visit friends   1. No, never 2. Yes, sometimes 3. Yes, often 4. No because of the pandemic (2021) | Which of the following activities do you usually do: Going to the movies, theatre, concerts, museums, exhibits   1. No, never 2. Yes, sometimes 3. Yes, often 4. No because of the pandemic (2021) |
| Which of the following activities do you usually do: Attend study circles   1. No, never 2. Yes, sometimes 3. Yes, often 4. No because of the pandemic (2021) | Which of the following activities do you usually do: Have friends over to visit   1. No, never 2. Yes, sometimes 3. Yes, often 4. No because of the pandemic (2021) | Which of the following activities do you usually do: Eating out at restaurants   1. No, never 2. Yes, sometimes 3. Yes, often 4. No because of the pandemic (2021) |
|  | Which of the following activities do you usually do: Visit relatives   1. No, never 2. Yes, sometimes 3. Yes, often 4. No because of the pandemic (2021) |  |
|  | Which of the following activities do you usually do: Have relatives over to visit   1. No, never 2. Yes, sometimes 3. Yes, often 4. No because of the pandemic (2021) |  |


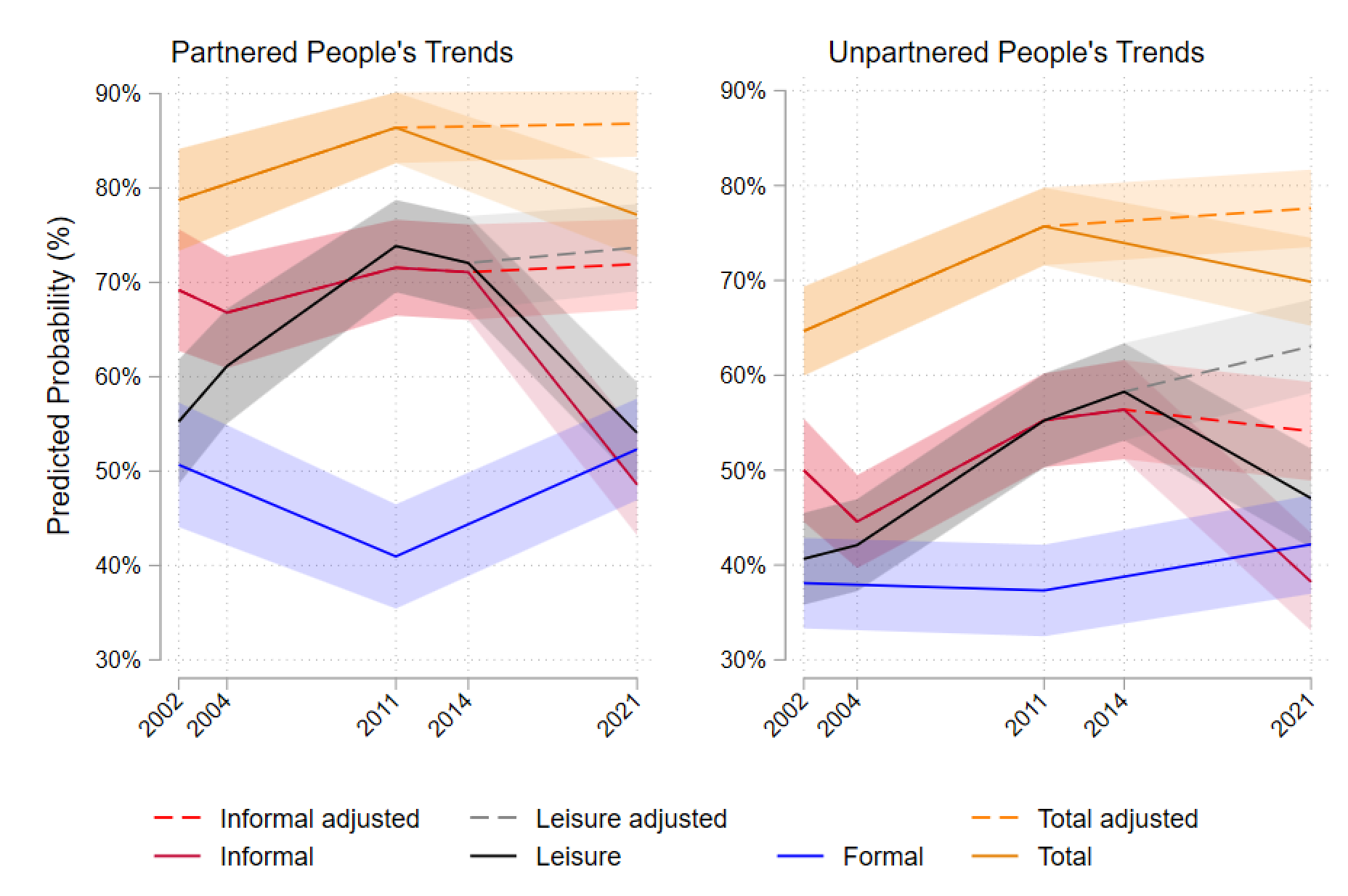


Figure S1. Predicted probabilities of social participation 2002-2021 age 77+ in Sweden by marital status. Partnered = Married or cohabiting with a partner, adjusted = adjusted for behavioural changes due to the COVID-19 pandemic.

| Table S2. Results from the KHB analysis with 2002 as baseline. | | | | | | | | | |
| --- | --- | --- | --- | --- | --- | --- | --- | --- | --- |
|  |  | Leisure | | Informal | | Formal | | Any | |
|  |  | OR | APE | OR | APE | OR | APE | OR | APE |
| 2004 | Reduced | 1.23 | 0.0412 | 0.801 | -0.0459 |  |  |  |  |
|  |  | [1.00,1.52] | [-0.00,0.08] | [0.64,1.01] | [-0.09,0.00] |  |  |  |  |
|  | Full | 1.089 | 0.0166 | **0.754** | **-0.0572** |  |  |  |  |
|  |  | [0.88,1.34] | [-0.02,0.06] | [0.60,0.95] | [-0.10,-0.01] |  |  |  |  |
|  | Diff | 1.13 | 0.0246 | 1.063 | 0.0113 |  |  |  |  |
|  |  | [0.88,1.46] | - | [0.85,1.33] | - |  |  |  |  |
|  | Confounding | Ratio | Percentage | Ratio | Percentage |  |  |  |  |
|  |  | 2.44 | 58.94 | .79 | -27.36 |  |  |  |  |
|  |  | Education | Function | Education | Function |  |  |  |  |
|  | %Mediated | 14.38 | 44.56 | -1.18 | -26.17 |  |  |  |  |
| 2011 | Reduced | **2.603** | **0.185** | **1.291** | **0.0506** | 0.891 | -0.0239 | **2.243** | **0.111** |
|  |  | [2.02,3.36] | [0.14,0.23] | [1.00,1.66] | [0.00,0.10] | [0.70,1.13] | [-0.07,0.02] | [1.66,3.03] | [0.07,0.15] |
|  | Full | **2.063** | **0.136** | 1.181 | 0.0321 | **0.750** | **-0.0594** | **1.808** | **0.0758** |
|  |  | [1.60,2.66] | [0.09,0.18] | [0.92,1.52] | [-0.02,0.08] | [0.59,0.95] | [-0.11,-0.01] | [1.34,2.44] | [0.04,0.11] |
|  | Diff | 1.262 | 0.0486 | 1.093 | 0.0185 | **1.188** | 0.0355 | **1.241** | 0.0355 |
|  |  | [0.98,1.63] | - | [0.88,1.37] | - | [1.01,1.39] | - | [1.03,1.50] | - |
|  | Confounding | Ratio | Percentage | Ratio | Percentage | Ratio | Percentage | Ratio | Percentage |
|  |  | 1.32 | 24.33 | 1.54 | 34.92 | .40 | -148.40 | 1.36 | 26.72 |
|  |  | Education | Function | Education | Function | Education | Function | Education | Function |
|  | %Mediated | 10.98 | 13.35 | 4.92 | 30.00 | -47.92 | -100.48 | 9.98 | 16.75 |
| 2014 | Reduced | **2.885** | **0.203** | **1.359** | **0.0605** |  |  |  |  |
|  |  | [2.20,3.78] | [0.15,0.25] | [1.04,1.78] | [0.01,0.11] |  |  |  |  |
|  | Full | **1.960** | **0.127** | 1.113 | 0.0208 |  |  |  |  |
|  |  | [1.50,2.56] | [0.08,0.18] | [0.85,1.46] | [-0.03,0.07] |  |  |  |  |
|  | Diff | **1.472** | 0.0758 | 1.222 | 0.0397 |  |  |  |  |
|  |  | [1.14,1.90] | - | [0.98,1.53] | - |  |  |  |  |
|  | Confounding | Ratio | Percentage | Ratio | Percentage |  |  |  |  |
|  |  | 1.57 | 36.49 | 2.88 | 65.22 |  |  |  |  |
|  |  | Education | Function | Education | Function |  |  |  |  |
|  | %Mediated | 16.62 | 19.87 | 7.08 | 58.39 |  |  |  |  |
| 2021 | Reduced | **3.253** | **0.223** | 1.266 | 0.0469 | **1.330** | **0.0594** | **2.344** | **0.116** |
|  |  | [2.50,4.23] | [0.18,0.27] | [0.98,1.64] | [-0.00,0.10] | [1.04,1.70] | [0.01,0.11] | [1.75,3.14] | [0.08,0.16] |
|  | Full | **1.592** | **0.0891** | 0.867 | -0.0286 | **0.762** | **-0.0560** | 1.173 | 0.0222 |
|  |  | [1.23,2.06] | [0.04,0.14] | [0.66,1.13] | [-0.08,0.02] | [0.59,0.98] | [-0.11,-0.00] | [0.87,1.58] | [-0.02,0.06] |
|  | Diff | **2.044** | 0.134 | 1.461 | 0.0755 | **1.745** | **0.115** | **1.999** | **0.0942** |
|  |  | [1.57,2.66] | - | [1.16,1.84] | - | [1.46,2.09] | - | [1.62,2.47] | - |
|  | Confounding | Ratio | Percentage | Ratio | Percentage | Ratio | Percentage | Ratio | Percentage |
|  |  | 2.54 | 60.59 | -1.65 | 160.67 | -1.05 | 195.24 | 5.34 | 81.28 |
|  |  | Education | Function | Education | Function | Education | Function | Education | Function |
|  | %Mediated | 29.30 | 31.29 | 18.33 | 142.34 | 64.71 | 130.53 | 31.18 | 50.09 |
|  | N | 3865 | 3865 | 3772 | 3772 | 2606 | 2606 | 2611 | 2611 |

| Table S3. Results from the KHB analysis with 2004 as baseline. | | | | | |
| --- | --- | --- | --- | --- | --- |
|  |  | Leisure | | Informal | |
|  |  | OR | APE | OR | APE |
| 2002 | Reduced | 0.813 | -0.0412 | 1.249 | 0.0459 |
|  |  | [0.66,1.00] | [-0.08,0.00] | [0.99,1.57] | [-0.00,0.09] |
|  | Full | 0.919 | -0.0166 | **1.327** | **0.0572** |
|  |  | [0.75,1.13] | [-0.06,0.02] | [1.05,1.67] | [0.01,0.10] |
|  | Diff | 0.885 | -0.0246 | 0.941 | -0.0113 |
|  |  | [0.69,1.14] | - | [0.76,1.16] | - |
|  | Confounding | Ratio | Percentage | Ratio | Percentage |
|  |  | 2.44 | 58.94 | .79 | -27.36 |
|  | %Mediated | Education | Function | Education | Function |
|  |  | 14.38 | 44.56 | -1.18 | -26.17 |
| 2011 | Reduced | **2.117** | **0.143** | **1.612** | **0.0965** |
|  |  | [1.67,2.68] | [0.10,0.19] | [1.28,2.04] | [0.05,0.14] |
|  | Full | **1.895** | **0.119** | **1.567** | **0.0893** |
|  |  | [1.49,2.40] | [0.08,0.16] | [1.24,1.98] | [0.04,0.14] |
|  | Diff | 1.117 | 0.024 | 1.029 | 0.00716 |
|  |  | [0.87,1.43] | - | [0.83,1.27] | - |
|  | Confounding | Ratio | Percentage | Ratio | Percentage |
|  |  | 1.20 | 14.79 | 1.06 | 5.96 |
|  | %Mediated | Education | Function | Education | Function |
|  |  | 10.04 | 4. 75 | 2.04 | 3.79 |
| 2014 | Reduced | **2.346** | **0.161** | **1.697** | **0.106** |
|  |  | [1.81,3.03] | [0.11,0.21] | [1.33,2.17] | [0.06,0.16] |
|  | Full | **1.800** | **0.110** | **1.476** | **0.0780** |
|  |  | [1.39,2.33] | [0.06,0.16] | [1.15,1.89] | [0.03,0.13] |
|  | Diff | **1.303** | **0.0512** | 1.15 | 0.0284 |
|  |  | [1.01,1.68] | - | [0.93,1.42] | - |
|  | Confounding | Ratio | Percentage | Ratio | Percentage |
|  |  | 1.46 | 31.05 | 1.36 | 26.36 |
|  | %Mediated | Education | Function | Education | Function |
|  |  | 17.17 | 13.88 | 3.54 | 22.39 |
| 2021 | Reduced | **2.645** | **0.182** | **1.581** | **0.0927** |
|  |  | [2.06,3.40] | [0.14,0.23] | [1.24,2.02] | [0.04,0.14] |
|  | Full | **1.462** | **0.0725** | 1.15 | 0.028 6 |
|  |  | [1.14,1.88] | [0.02,0.12] | [0.90,1.48] | [-0.02,0.08] |
|  | Diff | **1.809** | **0.11** | **1.375** | **0.0642** |
|  |  | [1.40,2.34] | - | [1.10,1.71] | - |
|  | Confounding | Ratio | Percentage | Ratio | Percentage |
|  |  | 2.56 | 60.94 | 3.28 | 69.52 |
|  | %Mediated | Education | Function | Education | Function |
|  |  | 32.47 | 28.47 | 8.87 | 60.65 |
|  | N | 3865 | 3865 | 3772 | 3772 |

| Table S4. Results from the KHB analysis with 2002 as baseline for women. | | | | | | | | | |
| --- | --- | --- | --- | --- | --- | --- | --- | --- | --- |
|  |  | Leisure | | Informal | | Formal | | Any | |
|  |  | OR | APE | OR | APE | OR | APE | OR | APE |
| 2004 | Reduced | **1.317** | **0.0548** | 0.755 | -0.0578 |  |  |  |  |
|  |  | [1.00,1.73] | [0.00,0.11] | [0.56,1.01] | [-0.12,0.00] |  |  |  |  |
|  | Full | 1.183 | 0.033 | **0.726** | **-0.0644** |  |  |  |  |
|  |  | [0.90,1.55] | [-0.02,0.09] | [0.54,0.98] | [-0.12,-0.01] |  |  |  |  |
|  | Diff | 1.113 | 0.0218 | 1.039 | 0.00664 |  |  |  |  |
|  |  | [0.80,1.54] | - | [0.78,1.39] | - |  |  |  |  |
|  | Confounding | Ratio | Percentage | Ratio | Percentage |  |  |  |  |
|  |  | 1.64 | 39.01 | .88 | -13.64 |  |  |  |  |
|  | %Mediated | Education | Function | Education | Function |  |  |  |  |
|  |  | 8.95 | 30.07 | -0.13 | -13.52 |  |  |  |  |
| 2011 | Reduced | **2.793** | **0.200** | 1.164 | 0.0302 | 0.886 | -0.0242 | **2.352** | **0.120** |
|  |  | [2.01,3.89] | [0.14,0.26] | [0.83,1.63] | [-0.04,0.10] | [0.65,1.21] | [-0.09,0.04] | [1.59,3.49] | [0.07,0.17] |
|  | Full | **2.189** | **0.149** | 1.069 | 0.013 | **0.722** | **-0.0654** | **1.844** | **0.0801** |
|  |  | [1.58,3.04] | [0.09,0.21] | [0.76,1.50] | [-0.05,0.08] | [0.53,0.99] | [-0.13,-0.00] | [1.25,2.72] | [0.03,0.13] |
|  | Diff | 1.276 | 0.0506 | 1.089 | 0.0171 | 1.228 | 0.0412 | 1.275 | 0.0397 |
|  |  | [0.92,1.77] | - | [0.81,1.46] | - | [0.99,1.53] | - | [0.99,1.64] | - |
|  | Confounding | Ratio | Percentage | Ratio | Percentage | Ratio | Percentage | Ratio | Percentage |
|  |  | 1.31 | 23.70 | 2.27 | 55.91 | .37 | -170.17 | 1.40 | 28.45 |
|  | %Mediated | Education | Function | Education | Function | Education | Function | Education | Function |
|  |  | 9.68 | 12.81 | 2.80 | 53.11 | -52.92 | -117.25 | 9.38 | 19.07 |
| 2014 | Reduced | **3.205** | **0.225** | 1.16 | 0.0295 |  |  |  |  |
|  |  | [2.24,4.59] | [0.16,0.29] | [0.81,1.66] | [-0.04,0.10] |  |  |  |  |
|  | Full | **2.084** | **0.140** | 0.949 | -0.0103 |  |  |  |  |
|  |  | [1.47,2.96] | [0.07,0.21] | [0.66,1.36] | [-0.08,0.06] |  |  |  |  |
|  | Diff | **1.538** | 0.0842 | 1.223 | 0.0398 |  |  |  |  |
|  |  | [1.11,2.14] | - | [0.91,1.65] | - |  |  |  |  |
|  | Confounding | Ratio | Percentage | Ratio | Percentage |  |  |  |  |
|  |  | 1.59 | 36.97 | -2.84 | 135.23 |  |  |  |  |
|  | %Mediated | Education | Function | Education | Function |  |  |  |  |
|  |  | 17.07 | 19.90 | 6.09 | 129.15 |  |  |  |  |
| 2021 | Reduced | **4.036** | **0.264** | 1.125 | 0.0234 | 1.163 | 0.0306 | **2.132** | **0.108** |
|  |  | [2.85,5.71] | [0.20,0.33] | [0.80,1.58] | [-0.04,0.09] | [0.84,1.62] | [-0.04,0.10] | [1.46,3.12] | [0.05,0.16] |
|  | Full | **1.952** | **0.128** | 0.799 | -0.0449 | **0.626** | **-0.0936** | 1.029 | 0.00418 |
|  |  | [1.39,2.74] | [0.06,0.19] | [0.56,1.13] | [-0.11,0.02] | [0.44,0.89] | [-0.16,-0.03] | [0.70,1.51] | [-0.05,0.06] |
|  | Diff | **2.067** | 0.136 | **1.408** | 0.0683 | **1.859** | 0.124 | **2.072** | 0.104 |
|  |  | [1.47,2.90] | [0.14,0.14] | [1.04,1.91] | [0.07,0.07] | [1.45,2.38] | [0.12,0.12] | [1.56,2.76] | [0.10,0.10] |
|  | Confounding | Ratio | Percentage | Ratio | Percentage | Ratio | Percentage | Ratio | Percentage |
|  |  | 2.08 | 52.05 | -0.52 | 291.48 | -0.32 | 410.54 | 26.25 | 96.19 |
|  | %Mediated | Education | Function | Education | Function | Education | Function | Education | Function |
|  |  | 26.17 | 25.88 | 14.35 | 277.13 | 155.03 | 255.52 | 38.35 | 57.84 |
|  | N | 2197 | 2197 | 2140 | 2140 | 1458 | 1458 | 1461 | 1461 |

| Table S5. Results from the KHB analysis with 2004 as baseline for women. | | | | | |
| --- | --- | --- | --- | --- | --- |
|  |  | Leisure | | Informal | |
|  |  | OR | APE | OR | APE |
| 2002 | Reduced | **0.760** | **-0.0548** | 1.325 | 0.0578 |
|  |  | [0.58,1.00] | [-0.11,-0.00] | [0.99,1.78] | [-0.00,0.12] |
|  | Full | 0.846 | -0.033 | **1.377** | **0.0644** |
|  |  | [0.64,1.11] | [-0.09,0.02] | [1.03,1.85] | [0.01,0.12] |
|  | Diff | 0.898 | -0.0218 | 0.962 | -0.00664 |
|  |  | [0.65,1.24] | - | [0.73,1.27] | - |
|  | Confounding | Ratio | Percentage | Ratio | Percentage |
|  |  | 1.64 | 39.01 | .88 | -13.64 |
|  | %Mediated | Education | Function | Education | Function |
|  |  | 8.95 | 30.07 | -0.13 | -13.52 |
| 2011 | Reduced | **2.121** | **0.145** | **1.543** | **0.0880** |
|  |  | [1.57,2.87] | [0.09,0.20] | [1.14,2.08] | [0.03,0.15] |
|  | Full | **1.851** | **0.116** | **1.473** | **0.0775** |
|  |  | [1.37,2.50] | [0.06,0.17] | [1.09,1.99] | [0.02,0.14] |
|  | Diff | 1.146 | 0.0288 | 1.048 | 0.0105 |
|  |  | [0.83,1.58] | - | [0.79,1.39] | - |
|  | Confounding | Ratio | Percentage | Ratio | Percentage |
|  |  | 1.22 | 18.11 | 1.12 | 10.77 |
|  | %Mediated | Education | Function | Education | Function |
|  |  | 10.66 | 7.45 | 0.90 | 9.87 |
| 2014 | Reduced | **2.434** | **0.170** | **1.538** | **0.0872** |
|  |  | [1.74,3.40] | [0.11,0.23] | [1.12,2.11] | [0.02,0.15] |
|  | Full | **1.762** | **0.107** | 1.307 | 0.0541 |
|  |  | [1.27,2.45] | [0.05,0.17] | [0.95,1.80] | [-0.01,0.12] |
|  | Diff | 1.382 | 0.0624 | 1.177 | 0.0331 |
|  |  | [1.00,1.91] | - | [0.89,1.56] | - |
|  | Confounding | Ratio | Percentage | Ratio | Percentage |
|  |  | 1.57 | 36.34 | 1.61 | 37.80 |
|  | %Mediated | Education | Function | Education | Function |
|  |  | 19.58 | 16.76 | 2.02 | 35.78 |
| 2021 | Reduced | **3.066** | **0.209** | **1.491** | **0.0811** |
|  |  | [2.20,4.27] | [0.15,0.27] | [1.09,2.05] | [0.02,0.15] |
|  | Full | **1.651** | **0.0955** | 1.1 | 0.0195 |
|  |  | [1.19,2.30] | [0.03,0.16] | [0.80,1.52] | [-0.05,0.09] |
|  | Diff | **1.857** | 0.114 | **1.355** | 0.0617 |
|  |  | [1.33,2.59] | - | [1.01,1.82] | - |
|  | Confounding | Ratio | Percentage | Ratio | Percentage |
|  |  | 2.23 | 55.26 | 4.20 | 76.19 |
|  | %Mediated | Education | Function | Education | Function |
|  |  | 30.40 | 24.85 | 4.14 | 72.05 |
|  | N | 2197 | 2197 | 2140 | 2140 |

| Table S6. Results from the KHB analysis with 2002 as baseline for men. | | | | | | | | | |
| --- | --- | --- | --- | --- | --- | --- | --- | --- | --- |
|  |  | Leisure | | Informal | | Formal | | Any | |
|  |  | OR | APE | OR | APE | OR | APE | OR | APE |
| 2004 | Reduced | 1.124 | 0.023 | 0.878 | -0.0271 |  |  |  |  |
|  |  | [0.81,1.56] | [-0.04,0.09] | [0.61,1.27] | [-0.10,0.05] |  |  |  |  |
|  | Full | 0.951 | -0.00964 | 0.783 | -0.0498 |  |  |  |  |
|  |  | [0.69,1.32] | [-0.07,0.05] | [0.54,1.13] | [-0.12,0.02] |  |  |  |  |
|  | Diff | 1.181 | 0.0326 | 1.121 | 0.0227 |  |  |  |  |
|  |  | [0.78,1.80] | - | [0.80,1.57] | - |  |  |  |  |
|  | Confounding | Ratio | Percentage | Ratio | Percentage |  |  |  |  |
|  |  | -2.32 | 143.17 | .53 | -87.99 |  |  |  |  |
|  | %Mediated | Education | Function | Education | Function |  |  |  |  |
|  |  | 37.96 | 105.22 | -9.29 | -78.70 |  |  |  |  |
| 2011 | Reduced | **2.400** | **0.164** | **1.535** | **0.0847** | 0.905 | -0.0214 | **2.097** | **0.0995** |
|  |  | [1.59,3.61] | [0.09,0.24] | [1.04,2.26] | [0.01,0.16] | [0.63,1.31] | [-0.10,0.06] | [1.32,3.33] | [0.04,0.16] |
|  | Full | **1.849** | **0.112** | 1.352 | 0.0577 | 0.774 | -0.0547 | **1.685** | **0.0646** |
|  |  | [1.23,2.78] | [0.04,0.19] | [0.92,1.99] | [-0.02,0.13] | [0.54,1.12] | [-0.13,0.02] | [1.06,2.68] | [0.01,0.12] |
|  | Diff | 1.298 | 0.0527 | 1.136 | 0.027 | 1.168 | 0.0333 | 1.245 | 0.035 |
|  |  | [0.85,1.98] | - | [0.81,1.60] | - | [0.93,1.47] | - | [0.94,1.66] | - |
|  | Confounding | Ratio | Percentage | Ratio | Percentage | Ratio | Percentage | Ratio | Percentage |
|  |  | 1.42 | 29.79 | 1.42 | 29.68 | .39 | -155.30 | 1.42 | 29.60 |
|  | %Mediated | Education | Function | Education | Function | Education | Function | Education | Function |
|  |  | 13.91 | 15.88 | 7.36 | 22.33 | -52.69 | -102.62 | 13.21 | 16.39 |
| 2014 | Reduced | **2.509** | **0.172** | **1.721** | **0.106** |  |  |  |  |
|  |  | [1.66,3.80] | [0.10,0.25] | [1.14,2.60] | [0.03,0.19] |  |  |  |  |
|  | Full | **1.809** | **0.108** | 1.41 | 0.0654 |  |  |  |  |
|  |  | [1.19,2.74] | [0.03,0.18] | [0.93,2.14] | [-0.01,0.14] |  |  |  |  |
|  | Diff | 1.387 | 0.064 | 1.22 | 0.0403 |  |  |  |  |
|  |  | [0.91,2.11] | - | [0.87,1.72] | - |  |  |  |  |
|  | Confounding | Ratio | Percentage | Ratio | Percentage |  |  |  |  |
|  |  | 1.55 | 35.56 | 1.58 | 36.70 |  |  |  |  |
|  | %Mediated | Education | Function | Education | Function |  |  |  |  |
|  |  | 15.71 | 19.85 | 6.43 | 30.27 |  |  |  |  |
| 2021 | Reduced | **2.432** | **0.167** | 1.484 | 0.0783 | **1.560** | **0.0955** | **2.713** | **0.127** |
|  |  | [1.62,3.65] | [0.09,0.24] | [0.99,2.22] | [-0.00,0.16] | [1.07,2.27] | [0.02,0.17] | [1.71,4.31] | [0.07,0.19] |
|  | Full | 1.192 | 0.0331 | 0.969 | -0.00636 | 0.966 | -0.00733 | 1.41 | 0.0442 |
|  |  | [0.79,1.79] | [-0.04,0.11] | [0.64,1.46] | [-0.09,0.08] | [0.66,1.41] | [-0.09,0.07] | [0.88,2.25] | [-0.02,0.10] |
|  | Diff | **2.041** | **0.133** | **1.532** | **0.0846** | **1.614** | **0.103** | **1.924** | **0.0829** |
|  |  | [1.32,3.15] | - | [1.08,2.18] | - | [1.25,2.09] | - | [1.40,2.64] | - |
|  | Confounding | Ratio | Percentage | Ratio | Percentage | Ratio | Percentage | Ratio | Percentage |
|  |  | 5.07 | 80.27 | -12.38 | 108.08 | -12.98 | 107.70 | 2.91 | 65.60 |
|  | %Mediated | Education | Function | Education | Function | Education | Function | Education | Function |
|  |  | 36.55 | 43.72 | 20.00 | 88.08 | 30.04 | 77.66 | 24.80 | 40.80 |
|  | N | 1668 | 1668 | 1632 | 1632 | 1148 | 1148 | 1150 | 1150 |

| Table S7. Results from the KHB analysis with 2004 as baseline for men. | | | | | |
| --- | --- | --- | --- | --- | --- |
|  |  | Leisure | | Informal | |
|  |  | OR | APE | OR | APE |
| 2002 | Reduced | 0.89 | -0.023 | 1.139 | 0.0271 |
|  |  | [0.64,1.24] | [-0.09,0.04] | [0.79,1.64] | [-0.05,0.10] |
|  | Full | 1.052 | 0.00964 | 1.277 | 0.0498 |
|  |  | [0.76,1.46] | [-0.05,0.07] | [0.88,1.84] | [-0.02,0.12] |
|  | Diff | 0.846 | -0.0326 | 0.892 | -0.0227 |
|  |  | [0.56,1.27] | - | [0.65,1.23] | - |
|  | Confounding | Ratio | Percentage | Ratio | Percentage |
|  |  | -2.32 | 143.17 | .53 | -87.99 |
|  | %Mediated | Education | Function | Education | Function |
|  |  | 37.96 | 105.22 | -9.29 | -78.70 |
| 2011 | Reduced | **2.136** | **0.141** | **1.748** | **0.112** |
|  |  | [1.45,3.14] | [0.07,0.21] | [1.20,2.54] | [0.04,0.19] |
|  | Full | **1.944** | **0.121** | **1.726** | **0.108** |
|  |  | [1.32,2.87] | [0.05,0.19] | [1.19,2.51] | [0.03,0.18] |
|  | Diff | 1.099 | 0.0201 | 1.013 | 0.00432 |
|  |  | [0.73,1.65] | - | [0.74,1.40] | - |
|  | Confounding | Ratio | Percentage | Ratio | Percentage |
|  |  | 1.14 | 12.39 | 1.02 | 2.32 |
|  | %Mediated | Education | Function | Education | Function |
|  |  | 10.22 | 2.17 | 3.48 | -1.16 |
| 2014 | Reduced | **2.233** | **0.149** | **1.960** | **0.133** |
|  |  | [1.49,3.35] | [0.07,0.22] | [1.32,2.90] | [0.06,0.21] |
|  | Full | **1.902** | **0.117** | **1.800** | **0.115** |
|  |  | [1.27,2.85] | [0.04,0.19] | [1.22,2.67] | [0.04,0.19] |
|  | Diff | 1.174 | 0.0314 | 1.089 | 0.0176 |
|  |  | [0.78,1.76] | - | [0.79,1.50] | - |
|  | Confounding | Ratio | Percentage | Ratio | Percentage |
|  |  | 1.25 | 19.96 | 1.14 | 12.63 |
|  | %Mediated | Education | Function | Education | Function |
|  |  | 12.48 | 7.47 | 3.40 | 9.23 |
| 2021 | Reduced | **2.165** | **0.144** | **1.689** | **0.105** |
|  |  | [1.47,3.19] | [0.07,0.21] | [1.15,2.49] | [0.03,0.18] |
|  | Full | 1.253 | 0.0428 | 1.237 | 0.0434 |
|  |  | [0.85,1.85] | [-0.03,0.12] | [0.84,1.83] | [-0.04,0.12] |
|  | Diff | **1.728** | 0.101 | 1.366 | 0.062 |
|  |  | [1.14,2.62] | - | [0.98,1.90] | - |
|  | Confounding | Ratio | Percentage | Ratio | Percentage |
|  |  | 3.42 | 70.79 | 2.47 | 59.50 |
|  | %Mediated | Education | Function | Education | Function |
|  |  | 36.34 | 34.45 | 12.74 | 46.76 |
|  | N | 1668 | 1668 | 1632 | 1632 |
